# From stereotyped recognition to situated meaning: university students’ retrospective accounts of perceived schema revision in learning Jiaxing revolutionary heritage

**DOI:** 10.3389/fpsyg.2026.1927045

**Published:** 2026-08-25

**Authors:** Xinsheng Jiang

**Affiliations:** School of Culture and Tourism, Jiaxing Vocational and Technical College, Jiaxing, China

**Keywords:** cross-cultural communication, cultural heritage education, historical empathy, prior knowledge, schema revision

## Abstract

This qualitative interview study examines how university students retrospectively narrate perceived schema revision in learning Jiaxing revolutionary heritage, a politically and culturally saturated local heritage context associated with South Lake and the Red Boat. Schema revision was used as a sensitizing analytical lens rather than as a direct measure of cognitive change. Thirty Mandarin interview transcripts from students with varied learning experiences were analyzed through a hybrid deductive–inductive codebook thematic analysis conducted by three coders. Participants commonly recalled exam-oriented, official-slogan, tourism/local-familiarity, professional-irrelevance, and cross-cultural-untranslatability schemas. They associated perceived meaning reconstruction with concrete people, local scenes, material artifacts, digital or visual media, mediating persons, and disciplinary entry points. Narrated reconstructions were often more humanized, contextualized, professionally relevant, and audience-aware, but remained partial and conditional. Evidence was strongest for changes in perceived relevance, interpretation, and explainability, not for verified real-time cognitive restructuring. The study therefore offers a provisional interpretive account of remembered learning pathways and design hypotheses for psychologically responsive, audience-sensitive heritage education.

## 1 Introduction

Revolutionary heritage education in Chinese higher education is not merely the transmission of historical facts. It is also a politically and culturally saturated learning context in which students interpret local symbols, historical narratives, and the contemporary significance of collective memory. Jiaxing, associated with South Lake, the Red Boat, and the closing session of the First National Congress of the Communist Party of China, provides a distinctive setting for examining these processes. Students encounter this heritage through courses, museum visits, digital exhibitions, volunteer interpretation, short videos, creative projects, and conversations with peers or international students. For educational and cultural psychology, the central question is how students connect such materials with prior knowledge and under what conditions they later describe the heritage as more specific, relevant, or explainable.

The study uses schema revision as a sensitizing lens. Schemas are organized knowledge structures that guide attention, interpretation, memory, and inference. Students do not approach Jiaxing revolutionary heritage as blank slates; they may carry textbook, examination, official-slogan, tourism, local-familiarity, professional-irrelevance, or cross-cultural-untranslatability schemas. These representations can support recognition while also reducing complex local history to labels, obligatory tasks, or politically charged phrases. Because the present evidence consists of retrospective interviews, however, the term perceived schema revision refers to participants’ narrated reorganization of meaning and is not treated as direct evidence of an underlying cognitive structure changing in real time.

This focus addresses a more specific gap than the broad claim that active heritage learning can promote engagement. Prior-knowledge research explains how existing representations shape attention and comprehension; historical-empathy research explains contextualized perspective taking; and museum and heritage studies show how objects, places, and participation can support meaning making. What remains less clear is how learners retrospectively connect these elements into a pathway from an identifiable prior representation to a changed relation among people, evidence, place, profession, and audience. The study advances this literature by (a) defining a conservative threshold for perceived schema revision, (b) retaining partial and resistant cases, and (c) separating direct cross-cultural encounters from hypothetical communication strategies. Jiaxing is therefore treated as a theoretically informative case rather than as evidence that one educational intervention necessarily produces cognitive change.

Accordingly, the study addresses four distinct research questions. RQ1: What prior representations of Jiaxing revolutionary heritage do students recall holding before their focal learning experiences? RQ2: Which situated encounters do participants identify as creating contrast, relevance, or renewed attention? RQ3: How do participants narrate the reorganization, persistence, or resistance of meaning after those encounters, and under what boundary conditions? RQ4: How do direct versus indirect or hypothetical cross-cultural experiences relate to participants’ willingness, framing strategies, and perceived—not demonstrated—capacity to explain the heritage to culturally different audiences? The interview guide and coding framework are provided in the Supplementary material.

## 2 Literature review

### 2.1 Schemas, prior knowledge, and knowledge revision

Schema theory provides the conceptual foundation for the study. In educational psychology, a schema is an organized knowledge structure that enables learners to recognize patterns, predict meaning, and integrate new information. Derry (1996) positions cognitive schema theory as a bridge between information-processing accounts and constructivist views of learning. From this perspective, learning is not the accumulation of isolated facts; it is an active process through which learners select, organize, and reinterpret information in relation to what they already know.

Memory research clarifies why schemas can both enable and constrain learning. Existing schemas support encoding and consolidation, but they can also promote selective attention, overgeneralization, and assimilation of new information into familiar categories (Ghosh and Gilboa, 2014; Gilboa and Marlatte, 2017; van Kesteren and Meeter, 2020). In the context of revolutionary heritage, a prior textbook schema may help students recall the historical status of the Red Boat, while the same schema may reduce the heritage to a set of examination answers. The double role of schemas makes revision a crucial analytic target.

Research on prior knowledge activation offers a more dynamic account of this process. Prior knowledge is not merely a background variable; it is activated, cued, and reorganized through discourse, tasks, and texts (Hattan and Alexander, 2020, 2021; Hattan et al., 2024; Hattan and Lupo, 2020). Background knowledge shapes comprehension and attention (Kaefer, 2020; Smith et al., 2021), while domain knowledge and comprehension develop reciprocally over time (Hwang et al., 2023). Prior knowledge can also influence engagement through cognitive load and help-seeking (Dong et al., 2020). These studies suggest that students’ engagement with heritage depends partly on whether their existing schemas are treated as resources, barriers, or objects of reflection.

Knowledge revision theory further specifies when change may occur. Kendeou (2024) argues that revision involves noticing inconsistency between existing knowledge and new information, evaluating competing representations, and updating a mental model. In this study, that theory provides a sensitizing account of possible change, while the interview data support only claims about participants’ remembered and narrated learning. A passage was therefore treated as evidence of perceived schema revision only when it included an identifiable prior representation, a concrete episode or contrast, and a reported reorganization of relationships or transfer into a new interpretation or explanatory strategy.

This operational threshold distinguishes perceived schema revision from adjacent outcomes. Knowledge gain denotes acquiring a new fact without reporting a changed organization of meaning; increased interest or emotion denotes motivational or affective response without interpretive reorganization; conceptual change usually concerns revision of a defined concept against disciplinary criteria; and narrative reframing denotes a new way of wording or plotting an account that may or may not reflect a broader change in representation. These processes can accompany perceived schema revision, but none was counted as sufficient on its own. Simple assent to interviewer language was likewise excluded unless supported by a situated example and reflective elaboration.

### 2.2 Historical meaning making and historical empathy

Revolutionary heritage learning is also a form of historical learning. Popa (2022) conceptualizes historical consciousness as meaning making: learners perceive the presence of the past in the present, interpret historical materials and narratives, and use historical understanding to orient themselves in contemporary life. Popa (2023) further describes a trajectory from noticing the past to interpreting it and applying historical understanding to current concerns. This trajectory is relevant to students who begin with static facts about the Red Boat and gradually ask how this local history matters to youth, profession, community, or communication.

Historical empathy research complements this account by showing that understanding the past involves cognitive perspective taking and affective engagement with historical actors’ situations, motives, constraints, and emotions (Karn, 2023). Such empathy can be developed through tasks that combine contextualization, perspective taking, and emotional involvement (Harnes, 2025). Witness narratives can support students’ understanding of empathy in history classrooms (Bartelds et al., 2023), and local history projects can promote historical empathy by placing inquiry in situated community contexts (Perrotta and Cross, 2024). These insights imply that Jiaxing heritage becomes meaningful when students encounter historical actors as situated persons rather than symbolic names.

The material and spatial dimensions of heritage learning also matter. Zachrich et al. (2020) argue that experiences with complex historical resources, including sites, artifacts, and witnesses, combine cognitive, emotional, and embodied dimensions. South Lake, museum exhibitions, original documents, digital reconstructions, family memories, and volunteer interpretation are therefore not interchangeable channels. They provide different affordances for attention, surprise, self-reference, and historical imagination.

Museum research further shows that material context and emotional engagement must be interpreted together rather than assumed to have automatic effects. Historical empathy in museums can emerge through the combination of contextualization and emotional engagement (Savenije and de Bruijn, 2017), while work on sensitive histories demonstrates that heritage resources can open inquiry but also reproduce discomfort, silence, or contested identification (Savenije et al., 2014). Community museums addressing difficult heritage likewise operate within social conflict and public memory rather than as neutral learning environments (Weiglhofer et al., 2023). These studies warrant caution when interpreting politically sensitive revolutionary heritage: an encounter with a site or object is an affordance for meaning making, not proof of a predetermined educational outcome.

### 2.3 Critical heritage, cultural education, and youth mediation

Cultural heritage education has increasingly been linked to identity, pride, and civic participation. Yu (2026) proposes a value-socialization model in which cultural heritage education relates to civic engagement through cognitive cultural identity and affective cultural pride. Wang et al. (2024) similarly associate cultural experience and interaction with cultural identity among ethnic Chinese youth, while Zhou et al. (2022) link red cultural atmospherics with red-education perception, red cultural identity, and confidence in Chinese culture. Prior learning and cultural identity can also shape university students’ learning preferences (Ma et al., 2025). Chinese-language scholarship has examined how digitized red-cultural resources may support university ideological and political education (Tian, 2022; Liu, 2023) and how red culture may be connected with university students’ striving spirit (Yi and Cheng, 2024). This work establishes the educational significance of the domain, but its largely normative or pathway-oriented emphasis leaves the micro-process and evidentiary threshold of learner-reported change comparatively underexplored.

Recent heritage research additionally emphasizes youth participation. Zhang et al. (2024) conceptualize youth participation as a component of cultural heritage management, and Miguel-Revilla et al. (2024) show that iconic heritage elements can serve as resources for history education. Developmental and identity research further suggests that emerging adulthood includes both stability and change in identity development (Branje et al., 2021). School-based interventions can support heritage identity when autonomy and relatedness are satisfied (Holscher et al., 2024), and narrative identity theory can illuminate experiences that students do not state directly (Tilley, 2025). Together, these literatures position students as potential interpreters, designers, translators, guides, or digital mediators rather than only as recipients.

A critical-heritage perspective complicates outcome-oriented accounts by asking whose meanings are authorized, how political and institutional narratives organize attention, and how audiences may negotiate rather than simply absorb heritage. Zhu (2024) argues that critical heritage studies must attend to knowledge produced beyond Anglophone settings while remaining reflexive about local power and epistemic conditions. This perspective is especially relevant to revolutionary heritage: official significance, local memory, pedagogical goals, professional interests, and audience interpretations may coexist without converging. Accordingly, respect, distance, curiosity, resistance, and selective relevance are treated here as analytically meaningful positions rather than as points on a single continuum of approval.

However, outcome-focused studies often leave the process and evidentiary status of perceived revision underspecified. The present study examines how prior representations, situated encounters, and later interpretations are connected in students’ retrospective narratives. It does not assume that these narratives transparently reveal underlying cognitive structures; instead, it asks what kinds of meaning reconstruction participants can articulate, where accounts remain partial or resistant, and how those accounts are shaped by disciplinary and communicative contexts.

### 2.4 Intercultural heritage communication and audience framing

Intercultural heritage communication involves more than replacing words across languages. Multilingual mediation shapes what visitors can notice, connect, and experience; for example, audio guides can organize heritage experience through selective narration and language-specific framing (Liao and Bartie, 2022). In politically sensitive heritage, terms may activate different historical and ideological associations across audiences. Audience-aware explanation therefore requires decisions about sequence, contextualization, analogy, and the relationship between local specificity and shared human experience.

The distinction between communication experience and communication imagination is crucial. Direct encounters may expose a learner to questions, misunderstanding, or feedback, whereas hypothetical responses show anticipated framing but cannot demonstrate communicative effectiveness. The analysis consequently separates participants who reported direct interaction, bilingual guiding, foreign-teacher questioning, or exchange-student discussion from those who responded only to indirect or hypothetical probes. RQ4 concerns perceived willingness and strategies in both groups, not verified effects on international audiences.

### 2.5 Analytical framework

The initial analytical framework comprised five deductive sensitizing domains: prior representations, situated encounters, cognitive-affective processing, possible revision or resistance, and reconstructed meaning. These domains informed broad interview coverage and first-cycle coding but were not treated as a template for final themes. Inductive codes retained participants’ language and unanticipated disciplinary, material, and audience-related patterns; candidate themes were developed by comparing these codes across cases and by testing them against partial-change and resistant accounts. The final themes therefore overlap with, but are not equivalent to, the sensitizing framework. This distinction preserved analytic openness and is documented in the supplementary coding framework.

## 3 Materials and methods

### 3.1 Design

This study adopted an interpretivist qualitative interview design. Semi-structured interviews were appropriate because the study sought participants’ retrospective constructions of subjective meaning, remembered turning points, affective responses, and perceived learning rather than direct measurements of cognitive change. Interview accounts were treated as situated interpretations produced in interaction with the interviewer; they can illuminate how participants now organize earlier experience, but they cannot establish the exact timing or causal sequence of psychological change (Alvesson, 2003). The design followed qualitative reporting standards in psychology and education (Levitt et al., 2018; O’Brien et al., 2014). The interview guide was piloted with three eligible students who were not included in the final corpus, and revisions reduced abstract wording while eliciting concrete episodes, people, places, objects, and artifacts.

### 3.2 Participants and data corpus

Participants were recruited through purposive maximum-variation sampling from a public higher vocational college in eastern China. Eligibility required at least one experience of learning about Jiaxing revolutionary heritage, including courses, museum visits, exhibitions, lectures, volunteer work, digital media, creative projects, field research, or cross-cultural communication. Variation was sought in year level, familiarity with Jiaxing, focal learning pathway, and disciplinary cluster. The analyzed sample comprised 30 students aged 18–22 years.

Recruitment was coordinated through student counselors in the institution’s constituent schools. Using the eligibility criterion above, counselors identified potentially eligible enrolled students and forwarded the study invitation. Of 46 students invited, 11 explicitly declined, most commonly because of coursework demands, internship scheduling conflicts, or no wish to discuss the topic. Thirty-five students commenced an interview; five voluntarily withdrew during its early stage, before a complete transcript was produced. These incomplete interviews were excluded, leaving 30 complete, usable Mandarin transcripts in the final corpus (P01–P30). Participation was entirely voluntary. No cash payment, gift or merchandise, course credit, comprehensive-assessment points, social-practice certification, or other material or academic incentive was provided.

To protect anonymity, individual majors and research directions are not linked to participant codes. Because disciplinary resonance became analytically important, Table 1 reports aggregated disciplinary clusters rather than identifiable individual majors. Discipline was not treated as a formal comparison variable. Instead, it was analyzed as a source of interpretive affordance: students used familiar disciplinary habits of seeing, reasoning, designing, caring, calculating, translating, or organizing to make sense of the same heritage materials.

**TABLE 1:** Aggregate description of the analyzed interview corpus.

Category	Description
Analytic corpus	Thirty usable Mandarin interview transcripts; all interviews were retained for analysis; participant codes P01–P30 were assigned during anonymization.
Age range	18–22 years.
Year level	First year: 4; second year: 10; third year: 12; fourth year: 4.
Disciplinary clusters	Humanities, languages, social sciences, and education: 10; design, media, and communication: 3; science, engineering, environment, food, and digital technology: 8; medicine and health-related fields: 2; business, finance, and management: 4; law and public administration: 3.
Familiarity with Jiaxing	Local or repeated everyday familiarity: 4; limited regional, travel, or occasional prior familiarity: 15; mainly textbook, digital, or first discipline-linked exposure before the focal learning experience: 11.
Dominant focal learning pathway	Course-led or self-guided museum/site learning: 7; digital or online exhibition/media-centered learning: 3; volunteer guiding, bilingual interpretation, or communication training: 5; creative, media, product, or curriculum design practice: 6; discipline-linked field research, specialized project, or professional lecture: 9.
Cross-cultural exposure	Direct cross-cultural interaction, bilingual guiding, foreign-teacher questioning, or exchange-student discussion: 11; indirect, hypothetical, online, or anticipated cross-cultural reflection only: 19.
Analytic focus	Initial schemas, triggering experiences, cognitive-affective processing, historical empathy, schema revision, reconstructed meaning, professional resonance, and perceived cross-cultural explainability.

Table 1 provides an aggregate overview of the analyzed corpus. Sample adequacy was judged by information power and thematic sufficiency rather than by treating saturation as a mechanical numerical threshold. The study had a focused aim, a specific eligible group, relatively long and information-rich interviews, planned variation across learning pathways and disciplines, and recurring patterns alongside negative and partial-change cases. Thirty transcripts therefore provided sufficient breadth and depth for the bounded interpretive claims made here, while not supporting statistical or cross-regional generalization (Guest et al., 2006; Hennink and Kaiser, 2022).

### 3.3 Data collection

Data were collected between February and April 2026 through online and face-to-face interviews in Mandarin, each lasting approximately 42–68 min. After informed consent, interviews were audio-recorded and transcribed verbatim. The interviewer explicitly welcomed indifference, distance, uncertainty, resistance, and limited or no change; reminded participants that there were no correct political or educational answers; and invited them to skip questions or stop the interview. Field notes documented context and preliminary reflections. The guide covered background experiences, recalled prior representations, situated encounters, cognitive-affective responses, historical empathy, personal meaning, cross-cultural interpretation, and suggestions for teaching and communication.

The interviews were conducted by the corresponding author, a full-time faculty member at the same institution. The interviewer had not taught, advised, supervised, graded, or otherwise assessed any participant and held no authority over participants’ course grades, practicum evaluations, or scholarship and award decisions. Before each interview, participants were explicitly informed of this non-evaluative relationship and that participation, withdrawal, and the content of their responses would have no bearing on any academic or institutional evaluation. This clarification was intended to reduce concerns about answering in ways they believed a teacher expected and to support candid responses.

Open narrative questions preceded specific reflective probes. Participants first described concrete episodes, people, places, objects, feelings, and perceived changes in their own words; examples such as movement from abstraction to concreteness were introduced only after an episode had been narrated. During analysis, simple agreement with interviewer-provided wording was not treated as evidence unless supported by a situated example and elaboration. Nevertheless, later probes may have prompted additional comparison or reinterpretation during the interview. The resulting accounts are therefore understood as co-constructed reflections on earlier learning, not transparent records of real-time cognition. Direct cross-cultural encounters were coded separately from indirect or hypothetical responses.

### 3.4 Coding and analysis

The primary analytical method was hybrid deductive–inductive codebook thematic analysis, using a collaboratively developed coding framework (Braun and Clarke, 2021, 2022; Byrne, 2022; Kiger and Varpio, 2020). Deductive sensitizing domains came from schema theory, knowledge revision, historical empathy, and heritage learning. They oriented attention but did not prescribe final themes. Inductive codes were developed from participants’ wording and recurrent patterns, including “professional hook,” “not against the content but against the form,” and “do not translate the concept first.” Reflexive memoing was used within this codebook approach to examine researchers’ assumptions and interpretive choices; the study does not claim to combine two distinct primary thematic-analysis methodologies. Iterative movement among transcripts, memos, codes, negative cases, and candidate themes supported conceptual model building (Naeem et al., 2023).

Three coders participated in the analysis. All three read the complete corpus and wrote familiarization and reflexivity memos. They then independently pilot-coded six transcripts selected to represent different learning pathways and compared segment boundaries, labels, and interpretations. For the remaining transcripts, a primary coder applied the revised codebook and at least one second coder checked the coding against the source text. All coders participated in consensus meetings and theme review. The purpose of comparison was interpretive refinement and an auditable application of definitions, not measurement of coder interchangeability; consequently, no intercoder-reliability coefficient was calculated.

After the codebook was revised, consensus meetings reviewed discrepant interpretations and negative cases, especially accounts of partial change or persistent distance. A passage counted as perceived schema revision only when it documented (a) a discernible earlier representation, (b) a concrete episode, contrast, or source of relevance, and (c) a reported reorganization of relations among historical actors, evidence, place, self, profession, or audience, or transfer into a new interpretive or explanatory strategy. New facts without reorganization were coded as knowledge gain; curiosity or emotion without reorganized meaning as interest/affect; and changed wording without a broader interpretive shift as narrative reframing. Indeterminate or interviewer-led assent was not counted. Codes were clustered into candidate themes, tested across the complete corpus, and revised through negative-case analysis. Participant coverage in the supplement documents breadth but is not a statistical frequency table.

### 3.5 Trustworthiness, reflexivity, and ethics

Trustworthiness was supported through pilot testing, repeated reading, codebook documentation, second-coder checking, consensus review, analytic and reflexivity memos, an audit trail, negative-case analysis, and translation checks. The team recognized that revolutionary heritage carries educational, cultural, political, and local meanings and that researchers working in the same institutional and regional context may normalize official narratives or overread local significance. Memos therefore recorded assumptions about heritage education, Jiaxing identity, disciplinary relevance, and cross-cultural communication and were revisited during coding and theme development. Social-desirability and institutional-power risks were addressed through voluntary consent, the absence of material or academic incentives, permission to skip or withdraw, explicit invitations to describe indifference or resistance, clarification that the faculty interviewer held no teaching or evaluative authority over participants, and the separation of simple assent from evidentiary coding. Nevertheless, the interviewer’s same-institution faculty status and counselor-mediated recruitment could not eliminate perceived institutional endorsement and are retained as limitations.

The study was reviewed and approved by the Ethics Review Committee of the School of Culture and Tourism, Jiaxing Vocational and Technical College, on 7 January 2026. Participants provided informed consent before the interviews and were informed of confidentiality, voluntary participation, the right to skip questions, and the right to withdraw. Recordings and transcripts were stored securely, and all identifying information was removed before analysis.

Quotations in the Findings are English translations of Mandarin interview excerpts. Candidate quotations were first selected in Mandarin for analytic fit, then translated into English and checked by a second coder against the original transcript. When a literal translation risked sounding unnatural, the team prioritized semantic accuracy while preserving the participant’s evaluative stance. Participant codes refer to anonymized transcripts.

## 4 Findings

Table 2 summarizes five analytically distinct but overlapping themes generated from the coded corpus. They are organized in a provisional sequence that mirrors the dominant structure of participants’ retrospective accounts; the order is not evidence of a universal temporal or causal process. Theme 1 addresses recalled prior representations (RQ1); Theme 2 identifies situated encounters (RQ2); Themes 3 and 4 examine narrated processing, reorganization, and resistance (RQ3); and Theme 5 distinguishes direct from indirect or hypothetical cross-cultural accounts (RQ4) while retaining boundary evidence relevant to RQ3. Participant codes indicate evidentiary breadth rather than frequency or effect size, and the excerpts preserve partial-change and resistant cases.

**TABLE 2:** Theme structure and evidentiary base.

Theme	Analytic claim	Illustrative participant codes
Theme 1: familiar yet distant initial schemas	RQ1 evidence: participants recalled exam, official-slogan, tourism, aesthetic, or professional-irrelevance representations that enabled recognition but sustained psychological distance.	P01–P30
Theme 2: concrete disruption of abstract labels	RQ2 evidence: participants identified concrete people, artifacts, archives, ecological sites, and spatial scenes as encounters that created contrast, relevance, or renewed attention.	P01, P03–P07, P11–P12, P15, P20, P22, P24, P26
Theme 3: disciplinary and self-referential routes	RQ3 evidence (and the processing component of RQ2): participants narrated disciplinary and self-referential routes to reorganization, but also selective or resistant responses.	P02–P04, P06, P08–P10, P13–P15, P17–P30
Theme 4: human mediation and participatory meaning	RQ3 evidence, with supporting relevance to RQ4: participants associated teachers, guides, peers, visitors, creators, and their own projects with a shift from reception toward interpretive participation.	P01–P02, P04–P05, P07, P11–P12, P15–P17, P20, P23, P28
Theme 5: bounded and audience-aware reconstruction	RQ4 evidence and RQ3 boundary evidence: participants reported more audience-aware framing, but direct and hypothetical cross-cultural accounts differed and neither verified audience effects.	P02–P05, P08–P10, P13–P16, P18, P21, P24, P26–P30

### 4.1 Theme 1: students recognized the heritage but often filed it under distant schemas

Most participants reported that they had known the Red Boat and South Lake before the focal learning experience, but recognition did not equal meaning. Initial schemas were commonly organized around examinations, official discourse, school tasks, tourism, or inherited local familiarity. P01 summarized an initial state as “I knew it was important, but it had nothing to do with me,” while P09 described an aesthetic stereotype as “red culture meant old-fashioned red-and-gold posters.” These short phrases capture a recurring pattern: the heritage was not unknown; it was over-known in a narrow form.

Local familiarity could produce distance rather than closeness. Several Jiaxing students had visited South Lake repeatedly since childhood, yet repetition without guided reinterpretation made the site feel ordinary. P06 explained:

*As a local student, I had been there so many times that it became a task point. We took a group photo, walked around for half an hour, and left. I had a little hometown pride, but mostly I was numb. It was famous, but it did not feel new, and I never thought it had anything to do with chemistry.*

This theme answers RQ1 by showing that initial schemas were not simply inaccurate. They were often useful for school assessment, city branding, or routine recognition, but they narrowed attention. Students looked for key terms, standard conclusions, or photo opportunities and often ignored less visible details such as archives, tools, ordinary people’s roles, and profession-specific dimensions. Distance could also be hidden behind respectful language: many participants affirmed the Red Boat’s historical importance while describing boredom, task completion, or limited relevance. This coexistence of recognition and distance is precisely what a schema approach can capture. It shows how meaning is organized, not merely whether attitudes are positive or negative.

### 4.2 Theme 2: participants recalled concrete encounters that disturbed abstract labels

Participants often located a remembered turning point in a concrete detail that conflicted with expectation. For some, the contrast concerned age: P01 recalled learning that one delegate was only 19 and said the history became related to “someone my age, not a distant great figure.” For others, handwritten corrections, a legal document, a medical kit, a cipher, an account book, or a logistics route map gave abstract history material specificity. These reports identify perceived contrasts in retrospect; they do not establish that the detail independently caused cognitive restructuring at the time.

P12’s account shows how local archival material transformed a macro-historical event into a situated local process:

*I had learned the First Congress mainly through central documents and representatives’ memoirs. In the local archive corner, I saw police records, boat-household records, and oral-history excerpts. Suddenly the transfer to South Lake was no longer a sentence in a textbook. It had railway times, local contacts, boatmen, public security conditions, and ordinary people who may not have known they were participating in a major historical moment.*

Participants did not always narrate contrast as an immediate emotional response. Surprise, curiosity, or professional interest sometimes preceded any reported empathy. Accounts were interpreted conservatively: phrases such as “I had not expected” or “I suddenly realized” indicated noticed contrast, but were counted as perceived revision only when the participant also described a changed relationship among facts, people, place, profession, or audience. Thus, a corrected manuscript, account book, ticket, child-oriented flag, or route map could function as more than an illustration in a participant’s account, while a newly remembered fact by itself remained knowledge gain rather than schema revision.

### 4.3 Theme 3: participants narrated disciplinary resonance as a route to relevance

A recurring cross-case pattern was that participants associated perceived reorganization with a route from heritage to familiar disciplinary or practical logic. Relevance often began as usability rather than civic identification. P06 was surprised that “revolutionary history actually had chemistry in it” after encountering invisible-ink principles. P15 reframed South Lake from a tourist task as “an ecological case with professional value” after noticing restoration and green-building details. P21 connected underground ciphers with mathematics and logic, and P24 interpreted early revolutionary legal documents as institutional design rather than slogans. These disciplinary hooks were narrated as cognitive entry points; they did not necessarily produce broader historical or civic meaning.

P18, a psychology student, explicitly interpreted the process through schema and empathy concepts:

*I used to think red education was just preaching and everyone was only coping with it. After the lecture, I realized that this was my own fixed schema. The teacher explained why concrete stories work: they activate prior schemas, create a little cognitive conflict, and make self-reference and historical empathy possible. It felt as if I had experienced the theory we were learning.*

For RQ2, these accounts identify disciplinary and self-referential relevance as conditions under which a situated encounter was noticed and elaborated. For RQ3, they show that perceived reorganization could remain selective: many participants still ignored content outside their professional or experiential entry point. Disciplinary resonance therefore functioned as a narrated bridge rather than a demonstrated endpoint. Evidence was strongest for changed relevance and interpretation; a stronger claim about cognitive restructuring would require longitudinal or real-time measures.

### 4.4 Theme 4: human mediation was associated with interpretive participation

Teachers, guides, visitors, peers, family members, digital creators, and student projects appeared repeatedly in accounts of perceived change. Participants contrasted passive exposure with dialogic or participatory experience. P07 recalled learning as a student guide that “guiding is not reciting a script; it is walking into history with the audience.” P11 revised a short-video script after meeting a retired guide and concluded that “it is not that red content has no audience; we had not told the right story.” P17 associated volunteer experience with seeing the museum as a public cultural service rather than a static propaganda site. These are participant-attributed associations, not estimates of mediator effects.

For bilingual and cross-cultural participants, human mediation was also training in audience perspective. P05 described the conflict between literal translation and meaning transmission:

*At first I translated the official script almost word for word. My teacher said foreign students would not understand it, so we rebuilt the story around Wang Huiwu and the young delegates. That was when I understood that translation is not word correspondence; it is meaning transmission. If the audience cannot enter the story, the most accurate terms are still ineffective.*

Participants who guided visitors, created videos, designed products, developed digital exhibits, built outdoor courses, or conducted field research described themselves less exclusively as recipients and more as mediators who selected evidence, adjusted narratives, and considered audiences. This reported repositioning supports the idea of students as human media. It also supplies RQ3 evidence because mediation required participants to articulate what they believed had become meaningful, and it supports RQ4 only at the level of self-reported framing practice or perceived capacity.

### 4.5 Theme 5: narrated reconstructions were more audience-aware but remained bounded

Narrated reconstructions tended to share three features. They were humanized when participants foregrounded young people, women, ordinary workers, family members, or unnamed local actors; contextualized when South Lake was connected with transport, space, local knowledge, or risk; and audience-aware when participants proposed beginning with a story, object, professional analogy, or historical comparison rather than with “red culture” or “Red Boat Spirit.” P16 proposed “a group of young Chinese people who wanted to make their country better,” and P29 said that “food is a language everyone understands.” These examples show reported framing choices, not tested audience reception.

Perceived revision remained selective. P04 said, “I still would not actively browse this content.” P14 reported that medical and nursing details would attract attention, P15 responded mainly to ecological and green-building details, and P03 described cognitive adjustment with limited emotional resonance. Such cases show why perceived schema revision cannot be equated with total attitude conversion: participants could reorganize a particular relation while retaining distance from compulsory formats, slogan-heavy texts, or content outside a disciplinary entry point.

Cross-cultural evidence was uneven. Eleven participants (P01, P05, P07, P12, P16, P17, P20, P22, P24, P26, and P30) reported direct interaction through bilingual guiding, foreign-teacher questioning, foreign-tourist contact, or exchange-student discussion. The other 19 participants (P02–P04, P06, P08–P11, P13–P15, P18–P19, P21, P23, P25, and P27–P29) offered only indirect, online, anticipated, or hypothetical responses. Both groups anticipated that a literal translation of “red culture” could activate political schemas before historical understanding; only the direct group, however, could describe an actual question, misunderstanding, or adjustment, and even those descriptions were retrospective self-reports.

*I would avoid the direct phrase red culture. I would say it is Jiaxing revolutionary historical heritage, then begin with a young woman, a group of young people, and a meeting that had to move because of danger. After the listener understands the story, we can talk about why the boat matters. If we start with the concept, they may only hear politics and stop listening.*

RQ4 is therefore answered at the level of perceived willingness and framing strategy, not demonstrated cross-cultural readiness. Direct-experience participants reported adjusting sequence, wording, analogy, or evidence in response to an audience, whereas hypothetical participants described what they expected they would do. Across both groups, audience awareness involved anticipating background knowledge and possible blocking terms. The study does not show that these strategies reduced misunderstanding or improved international audience outcomes; that claim requires observation and audience-based evaluation.

**FIGURE 1:**
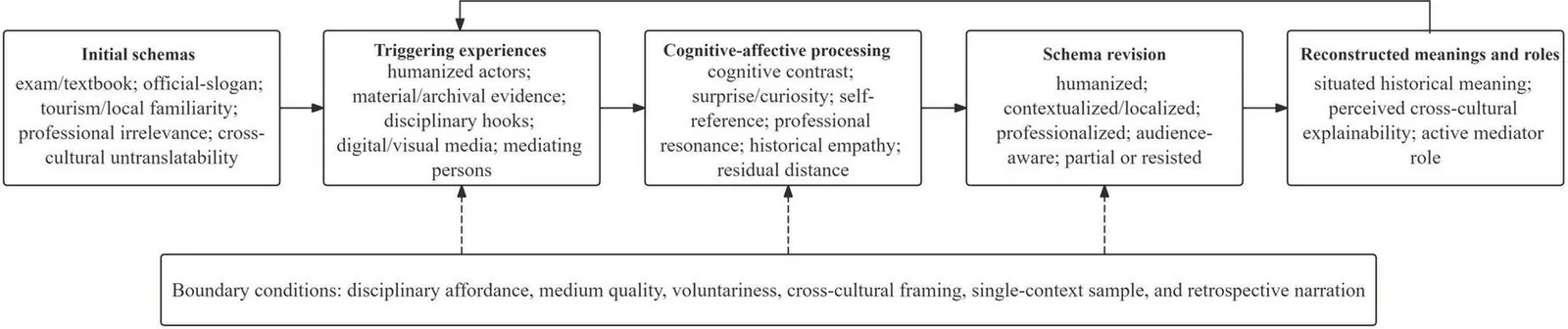
Provisional interpretive model of participants’ retrospective accounts of perceived schema revision in Jiaxing revolutionary heritage learning. The model summarizes recurrent relations described across interviews, not a validated causal or temporal process. Situated encounters were narrated as consequential when participants also reported contrast, self- or disciplinary relevance, historical imagination, or resistance. Perceived reconstructions were often humanized, contextualized, professionally relevant, and audience-aware, while boundary conditions and mediator roles shaped how participants now explained earlier learning.

Figure 1 offers a provisional interpretive model of how participants retrospectively organized these accounts. Its arrows summarize an analytic ordering among recalled prior representations, situated encounters, processing, and perceived reconstruction; they do not represent a validated temporal sequence or causal psychological mechanism.

## 5 Discussion

### 5.1 Schema revision as differentiation rather than simple attitude change

The main contribution of this study is a bounded interpretive account of how students retrospectively narrate movement from stereotyped recognition toward situated meaning. Across interviews, participants linked concrete evidence, self- or disciplinary relevance, historical empathy, and the need to re-express meaning for an audience with perceived reorganization. The evidence most clearly supports changes in relevance, interpretation, and reported explanatory framing. It is consistent with—but does not verify—schema differentiation or restructuring. This cautious interpretation aligns with research showing that prior knowledge can scaffold and constrain learning (Derry, 1996; Ghosh and Gilboa, 2014; Gilboa and Marlatte, 2017; van Kesteren and Meeter, 2020) and with Kendeou’s (2024) account of revision following noticed inconsistency and evaluation.

The accounts also extend prior-knowledge research by showing how familiar representations can operate as both barriers and entry points in a culturally saturated domain. Participants associated disciplinary, local, or personal knowledge with reduced distance and greater interpretive relevance (Dong et al., 2020; Hattan and Alexander, 2020, 2021; Hattan et al., 2024; Kaefer, 2020; Smith et al., 2021). Repeated exposure alone was not sufficient in their narratives: several local students recalled visiting the same sites many times without reporting deeper meaning until a task, detail, or mediator redirected attention. This finding is best understood as a design hypothesis—make prior representations visible and test whether strategically selected evidence supports reorganization—rather than as proof that any specific instructional technique will do so.

### 5.2 Historical empathy through specificity, materiality, and self-reference

Participants associated historical empathy with specificity rather than with abstract slogans. Age, handwriting, family ties, tools, archives, professional procedures, and embodied scenes helped them describe historical actors as situated persons. This pattern is compatible with historical consciousness as meaning making across past and present (Popa, 2022, 2023), historical empathy as contextualized cognitive-affective perspective taking (Karn, 2023; Harnes, 2025), and museum research that joins contextualization with emotional engagement (Savenije and de Bruijn, 2017). It also echoes work on eyewitnesses, local inquiry, and sensitive heritage, while retaining the possibility of discomfort or resistance (Bartelds et al., 2023; Perrotta and Cross, 2024; Savenije et al., 2014).

Materiality mattered because objects and environments made historical actors and conditions imaginable. A handwritten letter, a legal text, a medical box, a ship structure, an ecological shoreline, or a food package narrowed the distance between present-day students and past situations. This finding is consistent with the view that complex historical resources generate experiences that combine cognition, affect, and sensory imagination (Zachrich et al., 2020). For practice, artifacts should not be treated as decorative evidence after the “real” lesson. In many cases, artifacts and situated environments were the psychological trigger of the lesson itself. The findings also suggest that historical empathy should not be equated with emotional intensity alone. Some students engaged historically through legal, financial, medical, engineering, environmental, or logistical reasoning. Their empathy was analytical and role-based: they imagined constraints, trade-offs, and decisions from a professional standpoint. This broadens historical empathy in heritage education by including disciplined perspective taking alongside affective identification.

### 5.3 Youth as human media and the psychology of explainability

In participants’ accounts, perceived reconstructions sometimes became resources for mediation. Students who created videos, guided visitors, translated narratives, designed products, conducted research, or built activities reported learning through explanation. This is consistent with research positioning youth as participants in heritage interpretation rather than passive recipients (Zhang et al., 2024; Miguel-Revilla et al., 2024). It also connects with identity research on emerging adulthood, autonomy, relatedness, and narrative identity (Branje et al., 2021; Holscher et al., 2024; Tilley, 2025). However, mediation evidence varied: direct cross-cultural interactions supplied self-reported instances of adjustment, whereas hypothetical answers demonstrated only anticipated strategy.

The findings also complicate identity-centered accounts. For many participants, the strongest reported outcome was not explicit cultural pride or civic commitment, but professional relevance, narrative confidence, or cross-cultural explainability. This does not contradict research linking heritage education with identity and pride (Wang et al., 2024; Yu, 2026; Zhou et al., 2022). Rather, it suggests that identity internalization is one pathway among several. In a university context, professional self-concepts and disciplinary practices may provide important routes into cultural meaning, especially for students who resist overtly normative education.

### 5.4 Boundary conditions

Several boundary conditions qualify the findings. Disciplinary affordance was both gateway and limit; professional hooks invited attention but could narrow meaning to what appeared useful. Medium quality and voluntariness also mattered in participants’ narratives: compulsory, slogan-heavy, or low-quality formats were associated with task-completion schemas, whereas interactive and concrete formats were described more favorably. Cross-cultural explainability depended on anticipated audience framing, but story-based entry points and professional analogies were participant proposals rather than tested solutions. Critical-heritage perspectives further caution that official significance, local memory, institutional authority, and learner interpretation need not converge (Zhu, 2024; Weiglhofer et al., 2023).

Further boundaries concern scope, institutional power, and data production. The corpus came from one public higher vocational college and included students with at least one Jiaxing-related learning experience. Recruitment through student counselors and interviewing by a faculty member at the same institution may have signaled institutional endorsement and encouraged socially desirable responding, even though participation was voluntary, no material or academic incentives were offered, and the interviewer held no teaching, advising, supervisory, grading, or other evaluative authority over participants. Moreover, retrospective interviews do not simply retrieve stable memories: participants reconstruct earlier learning in the present interaction, and reflective probes may stimulate new comparison or meaning during the interview (Alvesson, 2003). The findings therefore concern remembered, narrated, and potentially co-constructed perceived revision—not real-time cognitive change. Future work should use longitudinal observation, pre/post conceptual tasks, stimulated recall, cross-regional samples, and international audience responses to test timing, mechanism, and communicative effects.

### 5.5 Educational and communication implications

The findings generate three design hypotheses for future testing. First, curricula could elicit prior representations before introducing concrete people, archives, tools, routes, or professional cases that create interpretable contrast. Second, heritage sites could compare layered pathways for general, disciplinary, and culturally different audiences rather than assume one script is universally accessible. Third, universities could evaluate participatory roles for guides, translators, creators, designers, researchers, and peer educators. Outcomes should include evidence use, historical contextualization, recognition of interpretive limits, and audience-sensitive explanation—not only reproduction of standard concepts. These proposals were not tested as interventions, and the present data do not establish policy effects, international communication success, economic value, or broader social impact; those applications require separate empirical evaluation.

### 5.6 Contribution to culturally situated educational psychology

The study contributes to culturally situated educational psychology by showing how a locally specific, politically saturated heritage case can refine questions about prior representation, meaning making, and mediation. The proposed relations may be relevant to other sites, monuments, museums, or civic narratives, but transferability depends on institutional history, political context, audience, and learning design. The Jiaxing accounts suggest that curiosity, professional logic, design interest, or explainability can precede identity affirmation; they do not establish a universal mechanism or eventual civic outcome.

The analysis also offers a provisional account of human mediation as psychological and communicative translation: turning a concept into a story, an object into a question, a professional method into an analogy, or a local memory into an audience-specific entry point. This account connects educational psychology with museum learning, heritage communication, and youth participation. Its next test is empirical: observe whether mediation tasks actually alter conceptual organization, historical contextualization, and audience understanding over time.

## 6 Conclusion

This study examined 30 university students’ retrospective accounts of learning Jiaxing revolutionary heritage. The confirmed qualitative finding is a recurrent narrated pattern: participants recalled recognizable but distant prior representations and associated greater specificity or relevance with young historical actors, local archives, material artifacts, disciplinary tasks, visual media, and dialogic mediation. Reported outcomes included humanized or contextualized interpretation, professional relevance, selective historical empathy, and audience-aware framing, alongside persistent distance and resistance. These findings describe perceived and narrated meaning reconstruction; they do not verify real-time schema restructuring.

The provisional model organizes these accounts around prior representations, situated encounters, cognitive-affective elaboration, perceived reconstruction, and boundary conditions. It should be read as an interpretive synthesis rather than a validated psychological sequence. Cross-cultural evidence is similarly qualified: direct-experience participants described self-reported adjustments, while most participants offered hypothetical strategies, and no international audience outcomes were measured.

Future research should test the model with longitudinal and multi-method designs, compare institutional and regional contexts, observe communication in practice, and include audience-based outcomes. Pending such tests, the practical recommendations are design hypotheses for eliciting prior representations, using specific evidence, supporting disciplinary entry points, and creating participatory mediation tasks—not claims of established policy, economic, international, or social effects.

## Data Availability

The raw data supporting the conclusions of this article will be made available by the author, without undue reservation.

## References

[B1] Alvesson M. (2003). Beyond neopositivists, romantics, and localists: A reflexive approach to interviews in organizational research. *Acad. Manag. Rev.* 28 13–33. 10.5465/AMR.2003.8925191

[B2] Bartelds H. Savenije G. M. van Drie J. van Boxtel C. (2023). Using eyewitnesses to promote students’ understanding of empathy in the history classroom. *J. Soc. Stud. Res.* 47 129–144. 10.1016/j.jssr.2022.12.001

[B3] Branje S. de Moor E. L. Spitzer J. Becht A. I. (2021). Dynamics of identity development in adolescence: A decade in review. *J. Res. Adolesc.* 31 908–927. 10.1111/jora.12678 34820948 PMC9298910

[B4] Braun V. Clarke V. (2021). One size fits all? What counts as quality practice in (reflexive) thematic analysis? *Qual. Res. Psychol.* 18 328–352. 10.1080/14780887.2020.1769238

[B5] Braun V. Clarke V. (2022). Conceptual and design thinking for thematic analysis. *Qual. Psychol.* 9 3–26. 10.1037/qup0000196

[B6] Byrne D. (2022). A worked example of Braun and Clarke’s approach to reflexive thematic analysis. *Qual. Quan.* 56 1391–1412. 10.1007/s11135-021-01182-y

[B7] Derry S. J. (1996). Cognitive schema theory in the constructivist debate. *Educ. Psychol.* 31 163–174. 10.1080/00461520.1996.9653264

[B8] Dong A. Jong M. S.-Y. King R. B. (2020). How does prior knowledge influence learning engagement? The mediating roles of cognitive load and help-seeking. *Front. Psychol.* 11:591203. 10.3389/fpsyg.2020.591203 33192933 PMC7658369

[B9] Ghosh V. E. Gilboa A. (2014). What is a memory schema? A historical perspective on current neuroscience literature. *Neuropsychologia* 53 104–114. 10.1016/j.neuropsychologia.2013.11.010 24280650

[B10] Gilboa A. Marlatte H. (2017). Neurobiology of schemas and schema-mediated memory. *Trends Cogn. Sci.* 21 618–631. 10.1016/j.tics.2017.04.013 28551107

[B11] Guest G. Bunce A. Johnson L. (2006). How many interviews are enough? An experiment with data saturation and variability. *Field Methods* 18 59–82. 10.1177/1525822X05279903

[B12] Harnes H. B. (2025). What can empirical research tell us about how to develop students’ historical empathy? A scoping review. *History Educ. Res. J.* 22:18. 10.14324/HERJ.22.1.18

[B13] Hattan C. Alexander P. A. Lupo S. M. (2024). Leveraging what students know to make sense of texts: What the research says about prior knowledge activation. *Rev. Educ. Res.* 94 73–111. 10.3102/00346543221148478

[B14] Hattan C. Alexander P. A. (2020). Prior knowledge and its activation in elementary classroom discourse. *Read. Writ.* 33 1617–1647. 10.1007/s11145-020-10022-8

[B15] Hattan C. Alexander P. A. (2021). The effects of knowledge activation training on rural middle school students’ expository text comprehension: A mixed methods study. *J. Educ. Psychol.* 113 879–897. 10.1037/edu0000623

[B16] Hattan C. Lupo S. M. (2020). Rethinking the role of knowledge in the literacy classroom. *Read. Res. Quar.* 55 S283–S298. 10.1002/rrq.350

[B17] Hennink M. Kaiser B. N. (2022). Sample sizes for saturation in qualitative research: A systematic review of empirical tests. *Soc. Sci. Med.* 292:114523. 10.1016/j.socscimed.2021.114523 34785096

[B18] Holscher S. I. E. Schachner M. K. Juang L. P. Altoe G. (2024). Promoting adolescents’ heritage cultural identity development: Exploring the role of autonomy and relatedness satisfaction in school-based interventions. *J. Youth Adolesc.* 53 2460–2479. 10.1007/s10964-024-02017-3 38789877 PMC11467014

[B19] Hwang H. McMaster K. L. Kendeou P. (2023). A longitudinal investigation of directional relations between domain knowledge and reading in the elementary years. *Read. Res. Quar.* 58 59–77. 10.1002/rrq.481

[B20] Kaefer T. (2020). When did you learn it? How background knowledge impacts attention and comprehension in read-aloud activities. *Read. Res. Quar.* 55 S173–S183. 10.1002/rrq.344

[B21] Karn S. (2023). Historical empathy: A cognitive-affective theory for history education in Canada. *Can. J. Educ.* 46 80–110. 10.53967/cje-rce.5483

[B22] Kendeou P. (2024). A theory of knowledge revision: The development of the KReC framework. *Educ. Psychol. Rev.* 36:44. 10.1007/s10648-024-09885-y

[B23] Kiger M. E. Varpio L. (2020). Thematic analysis of qualitative data: AMEE Guide No. 131. *Med. Teach.* 42 846–854. 10.1080/0142159X.2020.1755030 32356468

[B24] Levitt H. M. Bamberg M. Creswell J. W. Frost D. M. Josselson R. Suarez-Orozco C. (2018). Journal article reporting standards for qualitative primary, qualitative meta-analytic, and mixed methods research in psychology: The APA Publications and Communications Board task force report. *Am. Psychol.* 73 26–46. 10.1037/amp0000151 29345485

[B25] Liao M.-H. Bartie P. (2022). Translating heritage: A study of visitors’ experiences mediated through multilingual audio guides in Edinburgh Castle. *J. Heritage Tour.* 17 283–295. 10.1080/1743873X.2021.1976786

[B26] Liu X. (2023). [Pathways for integrating red cultural resources into the informatization of ideological and political education in higher education]. *Stud. Ideol. Educ.* 102–106.

[B27] Ma C. McKeown S. Rose J. (2025). Exploring influences of past learning experiences, individualist-collectivist cultural identity and social value orientations on Chinese and UK undergraduates’ learning preferences. *Front. Psychol.* 16:1555675. 10.3389/fpsyg.2025.1555675 40625443 PMC12231478

[B28] Miguel-Revilla D. Lopez-Torres E. Ortuno-Molina J. Molina-Puche S. (2024). Cultural heritage and iconic elements for history education: A study with primary education prospective teachers in Spain. *Human. Soc. Sci. Commun.* 11:1632. 10.1057/s41599-024-04123-w

[B29] Naeem M. Ozuem W. Howell K. Ranfagni S. (2023). A step-by-step process of thematic analysis to develop a conceptual model in qualitative research. *Intern. J. Qual. Methods* 22 1–18. 10.1177/16094069231205789

[B30] O’Brien B. C. Harris I. B. Beckman T. J. Reed D. A. Cook D. A. (2014). Standards for reporting qualitative research: A synthesis of recommendations. *Acad. Med.* 89 1245–1251. 10.1097/ACM.0000000000000388 24979285

[B31] Perrotta K. A. Cross K. (2024). Promoting historical empathy with a local history research project about the pandemic. *Soc. Stud. Res. Pract.* 19 171–190. 10.1108/SSRP-10-2023-0060

[B32] Popa N. (2022). Operationalizing historical consciousness: A review and synthesis of the literature on meaning making in historical learning. *Rev. Educ. Res.* 92 171–208. 10.3102/00346543211052333

[B33] Popa N. (2023). How meaning making cultivates historical consciousness: Identifying a learning trajectory and pedagogical guidelines to promote it. *Soc. Stud.* 114 139–159. 10.1080/00377996.2022.2140641

[B34] Savenije G. M. de Bruijn P. (2017). Historical empathy in a museum: Uniting contextualisation and emotional engagement. *Intern. J. Heritage Stud.* 23 832–845. 10.1080/13527258.2017.1339108

[B35] Savenije G. M. van Boxtel C. Grever M. (2014). Learning about sensitive history: “Heritage” of slavery as a resource. *Theory Res. Soc. Educ.* 42 516–547. 10.1080/00933104.2014.966877

[B36] Smith R. Snow P. Serry T. Hammond L. (2021). The role of background knowledge in reading comprehension: A critical review. *Read. Psychol.* 42 214–240. 10.1080/02702711.2021.1888348

[B37] Tian S. (2022). [Value and pathways of digitally empowering ideological and political courses with red cultural resources]. *J. Ideol. Theoret. Educ.* 2022 155–159. 10.16580/j.sxlljydk.2022.07.006

[B38] Tilley M. (2025). The things students don’t say: Theorising narrative identity to understand non-traditional students’ experiences in higher education. *Aus. Educ. Res.* 52 607–625. 10.1007/s13384-024-00732-1

[B39] van Kesteren M. T. R. Meeter M. (2020). How to optimize knowledge construction in the brain. *NPJ Sci. Learn.* 5:5. 10.1038/s41539-020-0064-y 32655882 PMC7339924

[B40] Wang Z. Ye S. Bei L. (2024). Leisure and cultural identity: An empirical study based on root-seeking summer camp for ethnic Chinese new generation. *Front. Psychol.* 15:1330613. 10.3389/fpsyg.2024.1330613 39539308 PMC11558882

[B41] Weiglhofer M. McCully A. Bates J. (2023). Learning about conflict: The role of community museums in educating on difficult heritage in a divided society. *Intern. J. Heritage Stud.* 29 365–381. 10.1080/13527258.2023.2189741

[B42] Yi D. Cheng L. (2024). [Value implications, main content, and practical pathways for cultivating university students’ striving spirit through red culture]. *J. Ideol. Theoret. Educ.* 146–151. 10.16580/j.sxlljydk.2024.06.009

[B43] Yu X. (2026). Cultural heritage education and civic engagement: A value-socialization model among Chinese university students. *Front. Psychol.* 17:1779128. 10.3389/fpsyg.2026.1779128 41835867 PMC12979467

[B44] Zachrich L. Weller A. Baron C. Bertram C. (2020). Historical experiences: A framework for encountering complex historical sources. *History Educ. Res. J.* 17 243–275. 10.14324/HERJ.17.2.08

[B45] Zhang Y. Ikiz Kaya D. van Wesemael P. J. V. Colenbrander B. J. F. (2024). Youth participation in cultural heritage management: A conceptual framework. *Intern. J. Heritage Stud.* 30 56–80. 10.1080/13527258.2023.2275261

[B46] Zhou X. Guo Y. Xie X. Liu C. Zhang F. (2022). The influence of a destination’s red cultural atmospherics on tourists’ confidence in Chinese culture. *Front. Psychol.* 13:992125. 10.3389/fpsyg.2022.992125 36051205 PMC9426675

[B47] Zhu Y. (2024). Putting the ‘critical’ in heritage studies from non-Anglophone regions: China and beyond. *Intern. J. Heritage Stud.* 30 1476–1486. 10.1080/13527258.2024.2393611

