# Supplementary material for: From stereotyped recognition to situated meaning: university students’ retrospective accounts of perceived schema revision in learning Jiaxing revolutionary heritage
Source: Front Psychol. 2026 Aug 25;17:1927045. doi: 10.3389/fpsyg.2026.1927045 (PMC13549911; doi:10.3389/fpsyg.2026.1927045)
Supplement: Supplementary file 1 [file Supplementary_file_1.docx]

# Supplementary Material A. Semi-Structured Interview Guide

## Opening script

Thank you for participating in this interview. This study focuses on how university students understand Jiaxing revolutionary heritage and whether they remember their understanding as changing through courses, visits, exhibitions, digital media, professional practice, or communication with others. There are no correct answers. We are equally interested in change, no change, uncertainty, indifference, resistance, and partial change. You may skip any question or stop the interview at any time.

Methodological note: The questions below are documented as the semi-structured guide used for data collection. Open narrative prompts preceded reflective probes. Because later questions may themselves have prompted comparison or reinterpretation, responses are analyzed as remembered and co-constructed accounts of perceived learning rather than as measurements of real-time cognitive change.

## A. Background and learning exposure

1. Please briefly introduce your year level, familiarity with Jiaxing, and previous contact with Jiaxing revolutionary heritage.

2. What learning experiences have you had related to Jiaxing revolutionary heritage, such as courses, visits to South Lake or the museum, exhibitions, lectures, volunteer work, student guiding, short videos, or communication with international students?

3. When you hear 'Jiaxing revolutionary heritage,' 'South Lake,' or 'the Red Boat,' what words, images, or feelings first come to mind?

## B. Initial schemas

4. Before your most recent or deepest learning experience, how did you understand revolutionary heritage? Did you mainly see it as textbook knowledge, exam content, political discourse, local history, tourism culture, moral education, or something else?

5. What emotional associations did you have at that time, such as familiarity, distance, interest, pride, pressure, indifference, confusion, or resistance?

6. Where did your earlier understanding mainly come from: textbooks, teachers, family, media, museums, short videos, peers, local community, travel, or other sources?

## C. Triggering learning experiences

7. Please describe one Jiaxing revolutionary heritage learning experience that impressed you deeply. What happened? Who was involved? Which place, object, story, image, or activity do you remember most clearly?

8. Was there a moment that surprised, moved, confused, challenged, or re-interested you? Please describe it concretely.

9. What role did real people play in this experience, such as teachers, guides, peers, volunteers, family members, historical actors, international students, or online creators?

## D. Cognitive-affective processing and schema revision

10. Did this experience change your understanding of Jiaxing revolutionary heritage? If so, how? If not, why not?

11. Looking back, how would you describe any change, no change, or partial change in your understanding? Please use your own words.

Probe, only after the participant has answered: Did anything become more concrete, more personally relevant, more story-based, more professionally meaningful, or easier or harder to explain to others?

12. Did you experience a conflict between old impressions and new experiences? What made you rethink?

13. Which parts of your earlier understanding did not change? Why?

14. Did you connect Jiaxing revolutionary heritage with your own life, learning, future work, civic responsibility, or student identity? How?

## E. Historical empathy and meaning making

15. Did any historical person, group, event, object, or place become easier to understand after learning? What helped you understand it?

16. When learning about historical actors, did you try to imagine their choices, emotions, constraints, or motives in their historical context? What helped or hindered this imagination?

17. What contemporary values or meanings do you now associate with Jiaxing revolutionary heritage? Which parts remain abstract or difficult to understand?

## F. Cross-cultural and international communication

18. Have you ever introduced, translated, discussed, or considered explaining Jiaxing revolutionary heritage to someone from a different cultural background? Please describe the situation. If the participant has no direct experience, ask as a hypothetical probe: If you had to introduce it to an international student or foreign friend, where would you begin, and why?

19. Which parts, if any, do you think might be difficult to explain across cultures? What words, stories, objects, comparisons, or frames would you use or avoid?

20. Have you discussed revolutionary heritage, Chinese history, or Jiaxing with people from different cultural backgrounds? What questions did they ask, and did those questions change your understanding?

## G. Closing questions

21. What teaching or communication methods could help university students move beyond stereotypes about revolutionary heritage?

22. Is there anything else you would like to add about your learning experience or your understanding of Jiaxing revolutionary heritage?

23. May we contact you later if clarification of the transcript is needed?

# Supplementary Material B. Coding Framework

B1. Coding process. The primary method was hybrid deductive–inductive codebook thematic analysis. Broad domains from schema theory, knowledge revision, historical empathy, and heritage learning functioned as deductive sensitizing concepts, not as a template for final themes. Inductive codes were developed from participants' wording and cross-case patterns. Reflexive memos documented researcher assumptions and interpretive decisions within the codebook workflow; reflexive thematic analysis was not treated as a second primary method.

Table B1. Coding process and audit trail

| **Stage** | **Description** |
| --- | --- |
| 1. Familiarization | All three coders read the complete corpus and wrote familiarization and reflexivity memos, including assumptions about revolutionary heritage, local identity, and cross-cultural communication. |
| 2. Pilot coding | All three coders independently coded six transcripts selected to represent different learning pathways and compared segment boundaries, labels, and interpretations. |
| 3. Codebook revision | The team refined definitions, inclusion and exclusion rules, examples, and decision rules for perceived schema revision; deductive domains remained sensitizing rather than determinative. |
| 4. Full coding | A primary coder applied the revised codebook to each remaining transcript; at least one second coder checked each transcript against the source text. |
| 5. Theme development | Candidate themes were developed from relationships among codes, compared across cases, and tested against partial-change and resistant accounts rather than mapped directly from the initial framework. |
| 6. Model integration | Themes were integrated into a provisional interpretive model of retrospective accounts; arrows were not treated as a validated causal or temporal sequence. |

B2. Participant coding status. Participant codes were assigned P01 through P30 during anonymization. All participant codes P01–P30 were coded and checked according to the same workflow. No transcript was excluded after anonymization.

Table B2. Participant coding status

| **Participant code** | **Coding status** |
| --- | --- |
| P01 | Coded |
| P02 | Coded |
| P03 | Coded |
| P04 | Coded |
| P05 | Coded |
| P06 | Coded |
| P07 | Coded |
| P08 | Coded |
| P09 | Coded |
| P10 | Coded |
| P11 | Coded |
| P12 | Coded |
| P13 | Coded |
| P14 | Coded |
| P15 | Coded |
| P16 | Coded |
| P17 | Coded |
| P18 | Coded |
| P19 | Coded |
| P20 | Coded |
| P21 | Coded |
| P22 | Coded |
| P23 | Coded |
| P24 | Coded |
| P25 | Coded |
| P26 | Coded |
| P27 | Coded |
| P28 | Coded |
| P29 | Coded |
| P30 | Coded |

B3. Codebook. The framework below was applied to all 30 transcripts. Participant coverage indicates cases with clear evidence and is not a frequency score or effect measure. Simple assent to interviewer wording was insufficient. Perceived revision required an identifiable prior representation, a concrete episode or contrast, and a reported reorganization of relations or transfer into a new interpretation or explanatory strategy. Knowledge gain, increased interest or emotion, and narrative reframing were coded separately unless this threshold was met.

Table B3. Coding framework and participant coverage

| **Code** | **Code family** | **Operational definition** | **Participant coverage** |
| --- | --- | --- | --- |
| A1 Exam/textbook schema | Initial schema | Heritage framed as content to memorize, answer, or test. | P01-P30 |
| A2 Official-slogan schema | Initial schema | Heritage associated with fixed political language, macro meaning, or one-way instruction. | P01-P30 |
| A3 Tourism/local-familiarity schema | Initial schema | Heritage treated as a city brand, scenic spot, routine visit, or familiar local landmark. | P01, P03, P06, P09, P11, P15, P17, P19, P23, P25, P29 |
| A4 Professional irrelevance schema | Initial schema | Heritage assumed to be unrelated to the learner's study, work, or disciplinary identity. | P02-P04, P06, P08-P10, P13-P15, P18-P30 |
| A5 Cross-cultural untranslatability schema | Initial schema | Participants anticipate that terms such as red culture or Red Boat Spirit will be misunderstood by foreign audiences. | P01-P05, P07, P09, P11-P13, P15-P18, P20, P22-P24, P26-P30 |
| B1 Humanized actors | Trigger | Concrete young people, women, ordinary residents, relatives, or unnamed workers make historical actors more person-like. | P01, P03-P07, P11-P12, P14, P17, P20, P22, P28-P29 |
| B2 Material and archival evidence | Trigger | Artifacts, manuscripts, account books, legal texts, medical tools, routes, models, ecological features, green-building systems, and archives disrupt abstract labels. | P01-P04, P06, P08, P10, P12, P14-P15, P19, P21-P22, P24, P26-P27, P29-P30 |
| B3 Disciplinary hook | Trigger | A professional or disciplinary entry point makes the heritage relevant and interpretable. | P02-P04, P06, P08-P10, P13-P15, P17-P30 |
| B4 Digital/visual/creative medium | Trigger | Digital exhibitions, short videos, design works, creative products, games, or visual narratives invite attention. | P02, P09, P11, P13, P19, P23, P25, P28-P29 |
| B5 Mediating person | Trigger | Teachers, guides, visitors, family members, peers, online creators, or staff explain, question, or model interpretation. | P01-P02, P04-P05, P07-P08, P10-P12, P14-P17, P20, P22-P24, P26-P30 |
| C1 Cognitive contrast | Processing | Participants notice a mismatch between prior expectation and situated evidence. | P01-P30 |
| C2 Self-reference and similarity | Processing | Age, gender, local identity, profession, or family memory lowers the distance to historical actors. | P01, P05, P07, P11-P12, P16, P18, P20, P22, P28 |
| C3 Professional resonance | Processing | Students interpret history through familiar disciplinary logic such as design, law, finance, medicine, engineering, environment, teaching, or logistics. | P02-P04, P06, P08-P10, P13-P15, P17-P30 |
| C4 Historical empathy | Processing | Participants imagine historical actors' constraints, motives, emotions, or situated choices. | P01, P03-P07, P11-P12, P16, P18, P20, P22, P25, P28 |
| C5 Residual resistance/distance | Processing | Participants maintain critique of compulsion, slogans, low-quality media, or weak personal relevance. | P02-P04, P06, P08-P10, P13-P15, P18, P21, P23-P24, P26-P30 |
| D1 Humanized revised schema | Perceived revision | Heritage reorganized around people, ordinary lives, and concrete stories rather than only abstract concepts. | P01, P04-P05, P07, P11, P20, P22 |
| D2 Contextualized/localized revised schema | Perceived revision | Historical events are understood as embedded in Jiaxing's spatial, social, ecological, and local conditions. | P01, P03, P12, P15, P17, P20 |
| D3 Professionalized revised schema | Perceived revision | Heritage becomes a professional case, design task, research object, or field-specific learning resource. | P02-P04, P06, P08-P10, P13-P15, P17-P30 |
| D4a Direct audience-aware framing | Perceived revision | A participant reports an actual cross-cultural interaction and describes an audience question, misunderstanding, or adjustment in sequence, wording, evidence, analogy, or frame. This is self-reported practice, not verified audience effect. | P01, P05, P07, P12, P16, P17, P20, P22, P24, P26, P30 |
| D4b Anticipated/hypothetical audience framing | Perceived strategy | A participant without direct interaction proposes how they might explain the heritage or anticipates audience difficulty. This supports perceived willingness or strategy only and is not counted as demonstrated communication practice. | P02-P04, P06, P08-P11, P13-P15, P18-P19, P21, P23, P25, P27-P29 |
| D5 Active mediator role | Perceived revision | Participant sees self as guide, translator, designer, researcher, creator, or peer educator. | P05, P07, P09, P11, P16-P17, P19, P23, P25, P28-P30 |
| E1 Medium-quality boundary | Boundary condition | Revision depends on concrete detail, interactivity, aesthetic quality, professional substance, and non-sloganized presentation. | P02, P04, P06, P08-P10, P13-P15, P18, P21, P23 |
| E2 Voluntariness boundary | Boundary condition | Compulsory visits, fixed reflection essays, or formalistic tasks limit curiosity and depth. | P02-P04, P06, P08, P10, P14-P15, P21, P25-P26 |
| E3 Cross-cultural framing boundary | Boundary condition | Directly translating red culture risks activating foreign audiences' political schemas before historical understanding. | P01-P05, P07, P09, P11-P13, P15-P18, P20, P22-P24, P26-P30 |
| E4 Study-context boundary | Boundary condition | Claims are limited by one regional higher-education context and by participants' prior Jiaxing-related learning exposure. The sample was intentionally varied in discipline, learning pathway, and familiarity with Jiaxing, but it was not designed for statistical or cross-regional generalization. | Corpus-level note |
| E5 Retrospective narration boundary | Boundary condition | Claims are based on participants' remembered and narrated learning episodes rather than real-time observation of cognitive change. Retrospective accounts can clarify perceived turning points and meaning making, but they cannot demonstrate the exact timing or causal sequence of schema revision. | Corpus-level note |
| E6 Interview-reactivity/co-construction boundary | Boundary condition | Open questions preceded probes, but reflective prompts may have generated additional comparison or reinterpretation during the interview. Accounts are treated as co-constructed present reflections on earlier learning, not transparent retrievals of a completed cognitive event. | Corpus-level note |

**Note:** E4 addresses institutional and sample scope; E5 addresses retrospective narration; and E6 addresses possible interview reactivity and co-construction. D4a and D4b keep direct cross-cultural experience analytically separate from anticipated or hypothetical framing.

# B4. Decision Rules for Perceived Schema Revision

The matrix records the conservative threshold used during consensus review. Adjacent outcomes could co-occur with perceived revision, but they were not sufficient by themselves.

| **Analytic label** | **Minimum evidence** | **Coding decision** |
| --- | --- | --- |
| Perceived schema revision | Identifiable prior representation + concrete episode/contrast + reported reorganization of relationships or transfer to a new interpretation/explanatory strategy. | Counted as perceived/narrated revision; never described as verified real-time cognitive restructuring. |
| Knowledge gain | A new fact, name, date, object, or detail is learned without a reported change in how meanings are related. | Coded as knowledge gain only unless the full revision threshold is also met. |
| Increased interest or emotion | Curiosity, pride, surprise, empathy, or other affect is reported without interpretive reorganization. | Coded as interest/affect only; emotional intensity is not treated as revision. |
| Narrative reframing | The participant adopts different wording, sequence, or story form without reporting changed relations among evidence, actor, place, self, profession, or audience. | Coded as reframing only unless broader reorganization or transfer is articulated. |
| Indeterminate/interviewer-led assent | The participant simply agrees with a probe or repeats interviewer language without a situated example and elaboration. | Not counted as evidence of revision. |
